# *HIST*ome2: a database of histone proteins, modifiers for multiple organisms and epidrugs

**DOI:** 10.1186/s13072-020-00354-8

**Published:** 2020-08-03

**Authors:** Sanket G. Shah, Tushar Mandloi, Pooja Kunte, Abhiram Natu, Mudasir Rashid, Divya Reddy, Nikhil Gadewal, Sanjay Gupta

**Affiliations:** 1 Epigenetics and Chromatin Biology Group, Gupta Laboratory, Cancer Research Institute, Advanced Centre for Treatment, Research and Education in Cancer, Tata Memorial Centre, Kharghar, Navi Mumbai, MH 410210 India; 2 Bioinformatics Centre, Advanced Centre for Treatment, Research and Education in Cancer, Tata Memorial Centre, Kharghar, Navi Mumbai, MH 410210 India; 3 Homi Bhabha National Institute, Training School Complex, Anushakti Nagar, Mumbai, MH 400085 India; 4 Present Address: Diabetes Unit, King Edward Memorial Hospital Research Centre, Rasta Peth, Pune, Maharashtra 411 011 India; 5 Present Address: Stowers Institute for Medical Research, Kansas City, MO 64110 USA

**Keywords:** Histones, PTMs, Enzymes, Epidrugs

## Abstract

**Background:**

Epigenetics research is progressing in basic, pre-clinical and clinical studies using various model systems. Hence, updating the knowledge and integration of biological data emerging from in silico, in vitro and in vivo studies for different epigenetic factors is essential. Moreover, new drugs are being discovered which target various epigenetic proteins, tested in pre-clinical studies, clinical trials and approved by the FDA. It brings distinct challenges as well as opportunities to update the existing *HIstome* database for implementing and applying enormous data for biomedical research.

**Results:**

*HISTome2* focuses on the sub-classification of histone proteins as variants and isoforms, post-translational modifications (PTMs) and modifying enzymes for humans (*Homo sapiens*), rat (*Rattus norvegicus*) and mouse (*Mus musculus*) on one interface for integrative analysis. It contains 232, 267 and 350 entries for histone proteins (non-canonical/variants and canonical/isoforms), PTMs and modifying enzymes respectively for human, rat, and mouse. Around 200 EpiDrugs for various classes of epigenetic modifiers, their clinical trial status, and pharmacological relevance have been provided in *HISTome2*. The additional features like ‘Clustal omega’ for multiple sequence alignment, link to ‘FireBrowse’ to visualize TCGA expression data and ‘TargetScanHuman’ for miRNA targets have been included in the database.

**Conclusion:**

The information for multiple organisms and EpiDrugs on a common platform will accelerate the understanding and future development of drugs. Overall, *HIST*ome2 has significantly increased the extent and diversity of its content which will serve as a ‘knowledge Infobase’ for biologists, pharmacologists, and clinicians. *HISTome2: The HISTone Infobase* is freely available on http://www.actrec.gov.in/histome2/.

## Background

The nucleosome is a fundamental unit of chromatin that plays an essential role in governing biological processes like gene expression, gene regulation, and DNA repair. Each nucleosome consists of ~ 147 base pairs of DNA wrapped around an octamer of histone proteins—a tetramer of H3/H4 and two dimers of H2A/H2B [1]. Histones are usually categorized into ‘canonical’ and ‘non-canonical’. Canonical histones, also defined as histone isoforms, are located in clusters at a gene level and are exclusively expressed in the S-phase of the cell cycle with 3’ stem-loop organization. Whereas non-canonical histones or histone variants are solitary genes, expressed in a replication-independent manner throughout the cell cycle with poly-A tail [2]. The isoforms vary among themselves only by a few amino acids, whereas variants significantly differ from canonical histones as well as among each other.

Structural shifts between euchromatin and heterochromatin take place by reversible incorporation of histone variants in the nucleosomes with site-specific post-translational modifications. Earlier studies have shown that the replacement of a canonical histone by a variant is a dynamic process and account for the complexity contributing to the cell fate and genome plasticity [3–6]. Recently, histone isoforms are also emerging as an important player in the field of chromatin biology and have shown to be functionally non-redundant [7]. Growing evidence suggests that aberrant regulation of gene expression through the incorporation of histone isoforms and variants, and their site-specific post-translational modifications are strongly associated with different human diseases like cancer, diabetes, adiposity, auto-immune diseases, Alzheimer’s disease [8–11]. The macroH2A levels were found to be decreased in lung cancer [12]. H2A.Z was also found to be associated with expression of cell proliferation and differentiation related genes [13, 14]. Further, the overexpression of H2A.Z.2 was found to be associated with poor survival in melanoma [15]. H3 variant CENP-A expression is also reported to increase in breast and colon cancer [16]. Further, the differential expression of histone-modifying enzymes is reported in various human diseases for site-specific histone modifications [17]. High levels of H4K5Ac, H3K27Ac, H3K18Ac, H4K8Ac are associated with lung and ovarian cancer [18, 19]. HATs like CREBBP (CREB binding protein) and EP300 were found to be over-expressed in the colon and small cell lung cancers [20, 21]. In parallel, HDAC 1, 2 and 3 were also reported to over-express in gastric, lung, breast and hepatocellular cancers [22]. Histone methyl transferase, EZH2 along with site-specific methylation, H3K27me3 were found to be upregulated in breast and prostate cancer [23]. Different kinases and ubiquitinases were also reported to be altered in cancer [24, 25]. In the last 15 years, a different class of drugs known as epidrugs has been developed that target the histone-modifying enzymes. Epidrugs have shown promising results in pre-clinical models as well as in clinical trials. In particular, new and specific DNMT and HDAC inhibitors were screened in cell lines and pre-clinical animal models of several cancers, such as breast, skin, colorectal and liver. The DNMT and HDAC inhibitors are also approved by the FDA for use in clinics against cancer and other diseases [26].

In the past decade, several databases related to different aspects of chromatin biology were developed due to the importance of histone proteins in the regulation of DNA-mediated cellular processes. The databases such as Human Histone Modification Database (HHMD) gives information about experimentally identified human histone PTMs [27], ChromDB provides details about histone modifications on *Saccharomyces cerevisiae* [28], Histone Systematic Mutation Database (HistoneHits) contains relevant data about histone mutants in yeast [29] and Histone Database aims to focus on histone structures and sequences in many species [30]. Also, the human epigenetic drug database (HEDD) focuses on epigenetic drugs obtained from experiments and curated data [31]. The earlier version of *the HI*stome database provides information about histone proteins, histone PTMs and its modifiers in humans [32]. The key importance of histones and associated functional proteins along with ever-growing information in multiple cellular functions, organisms, and diseases is necessary to update the current database, *HI*stome: The Histone Infobase.

In light of the above needs, *the HIstome* database is updated to *HISTome2: The Histone Infobase,* which is available at http://www.actrec.gov.in/histome2/. The new Infobase has been created, which covers information on histone proteins, variants, and isoforms separately; their sites of modifications and modifying enzymes from mammalian systems such as *Homo sapiens, Rattus norvegicus,* and *Mus musculus*. Further, to visualize RNA expression of human epigenetic enzymes and histone proteins in cancer, a link to TCGA FireBrowse has been included [33]. A list of all putative miRNAs for all human entries has been provided via TargetScan [34]. The literature for all the entries is made available by connecting each entry with PubMed. Similarly, our new version of the database introduces detailed information about epidrugs and their ongoing experimental, pre-clinical and clinical studies. The EpiDrug database entries have been categorized by the classes of histone- or nucleic acid-modifying enzymes with their known inhibitors like HATi, HDACi, DNMTi, etc. Further, to enhance the utility of database for the scientific community, new features such as multiple sequence alignment (MSA), histone isoform dimer stability prediction by energy minimization, and advanced search have been integrated to extract query-based information. MSA will help in understanding the sequence similarities and conservation of histone proteins across species. Further, the difference in energy minimization of highly conserved histone dimers might get reflected on nucleosome stability.

Overall, the updated version of the histone database has significantly increased the extent, diversity of its content and thus assisting in the search and comparative interpretation of the multifactorial parameters in the field of histone biology. The comprehensive information available on the database will serve as a useful “knowledge base” for fundamental researchers, clinical scientists, pharmacologists in understanding the histone biology and their potential importance in epidrug discovery through its ability to connect chemistry, biology, and informatics.

## Results: updates and new features

### Home page for an integrative search for multiple organisms, EpiDrug, and tools

**Fig. 1:**
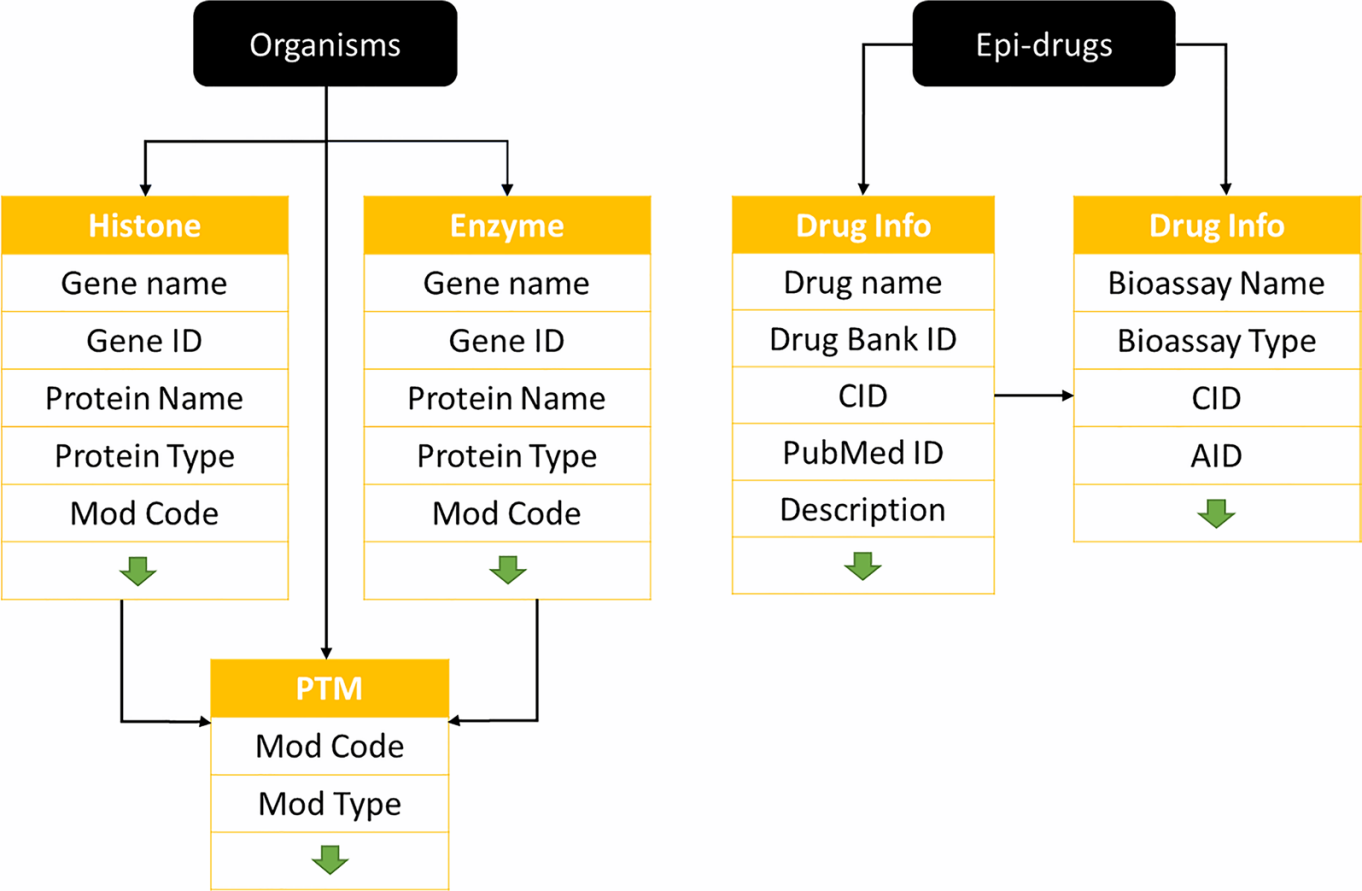
Schematic representation of the *HISTome2* database: the information related to the different organisms, *Homo sapiens, Rattus norvegicus, and Mus musculus* is stored in tables like Histone, PTM, and Enzyme. Histone, PTM, and Enzyme table are linked by ‘Mod Code’ and EpiDrug contains Drug info and bioassay tables which are linked by ‘CID’

The new version, *HISTome2*, has been designed to integrate the information of human, mouse, rat and EpiDrug database by a user-friendly interface. The database home page consists of two bars viz. navigation and menu bar. The schema of database is mentioned in construction part of methods section (Fig. 1). The navigation bar helps the user to navigate to the individual databases for human, mouse, rat, and EpiDrug. The menu bar helps the user to retrieve information about the variants, isoforms, PTMs, writers, and erasers for all organisms. For example, upon bringing the cursor over variants, isoforms, writers, and erasers, a drop-down menu will appear whereby with a single click, the user can retrieve the information of all the three organisms at a time. However, for PTMs, clicking on respective modifications, common as well as a unique site of modification will be displayed. Also, the video tutorial has been included under ‘how to use’ for easy navigation through the database.

### New inclusion and updation of the entries

**Fig. 2:**
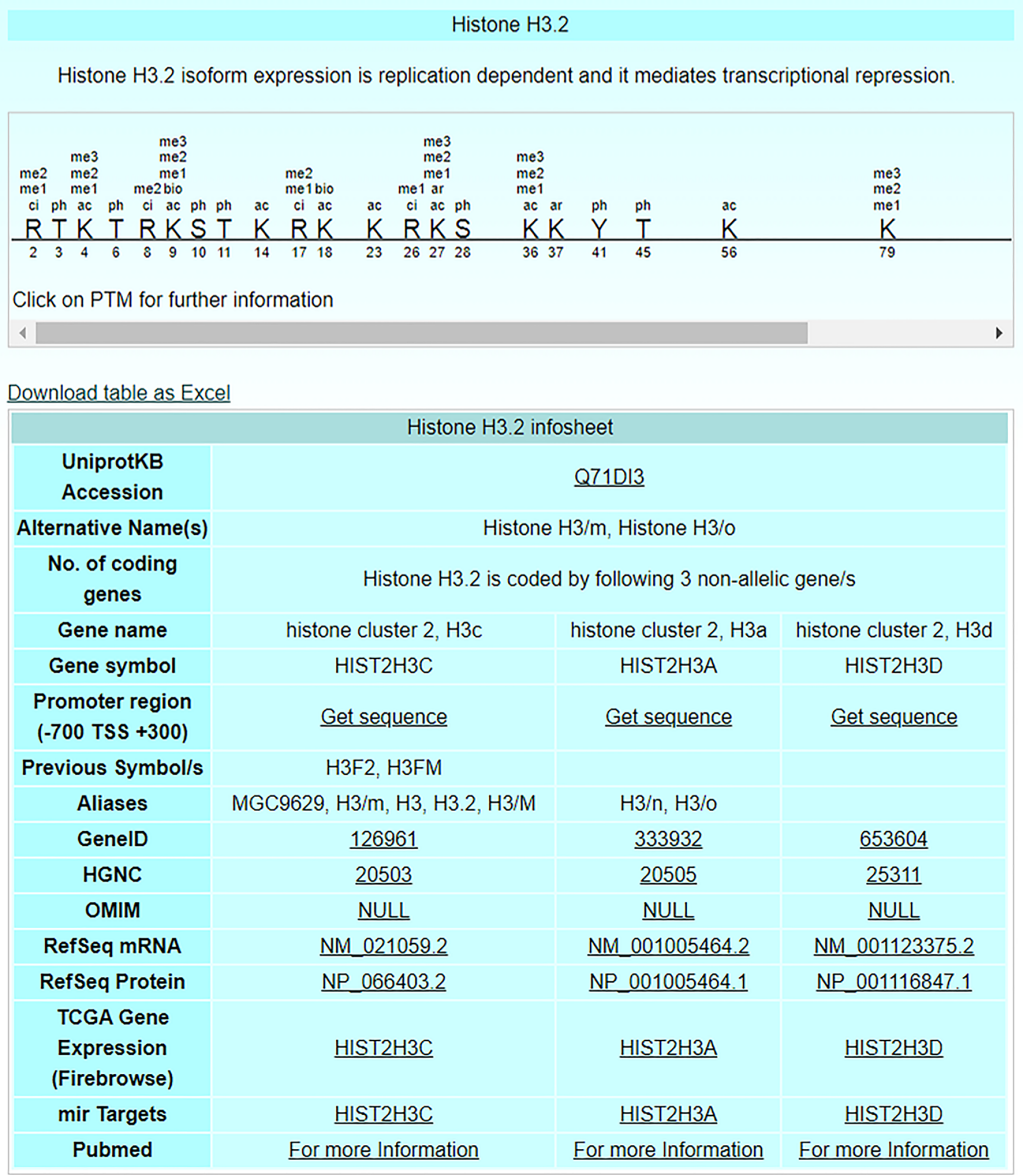
Representative image of histone entry page for Human: The individual entry has brief write-up for biological role, site-specific modifications and different content which are divided into uniport ID, synonym, number of coding genes, gene name, gene symbol, promoter region, gene ID, HGNC, Refseq mRNA, Refseq protein, TCGA expression and mIR targets for a specific histone

In the updated version, the information for the histone proteins is subcategorized as canonical and non-canonical. These histone proteins, their PTMs and modifying enzymes for human, mouse, and rat are included in the MySQL database. An earlier version of the *HI*stome database contained information of 55 histone proteins, 106 PTMs, and 152 modifying enzymes only for human database [32]. In the updated version *HISTome2*, the histone proteins for humans contain 97 entries with a sub-classification of 33 variants (non-canonical) and 64 isoforms (canonical), 114 distinct sites of histone modifications, and 161 modifying enzymes. The new information for routinely used pre-clinical model systems, rat and mouse is included in the *HISTome2*. Rat database carries 48 entries of histone proteins with sub-division of 21 variants (non-canonical), and 27 isoforms (canonical), 61 distinct sites of their modifications and 89 modifying enzymes. Mouse database has 87 entries of histone proteins which are sub-categorized into 26 variants (non-canonical) and 61 isoforms (canonical), 92 distinct sites of their modifications and 100 modifying enzymes. To each entry of histone proteins and modifying enzymes in the database, a ‘detailed information page’ is developed, which gives hyperlinks to external databases for further information (Fig. 2). Further, to gain the real-time updated references of these entries, a link to PubMed is provided with pre-embedded keywords.

The differential gene expression of histone variants, isoforms, and epigenetic modifiers has been reported in various pathophysiological conditions like cancer. The information related to altered expression of histone variants, isoforms, and epigenetic modifiers in human is available on TCGA (The Cancer Genome Atlas) databases [35]. Therefore, *the HISTome2* database provides a link to TCGA FireBrowse from which expression profiles of different histones in normal and cancer of various tissue types in humans can be extracted [33]. The expression of histone genes and modifying enzymes is also regulated by microRNAs; therefore, a link to the TargetScan database is provided to extract the probable microRNAs that can regulate expression of specific target genes [34].

**Fig. 3:**
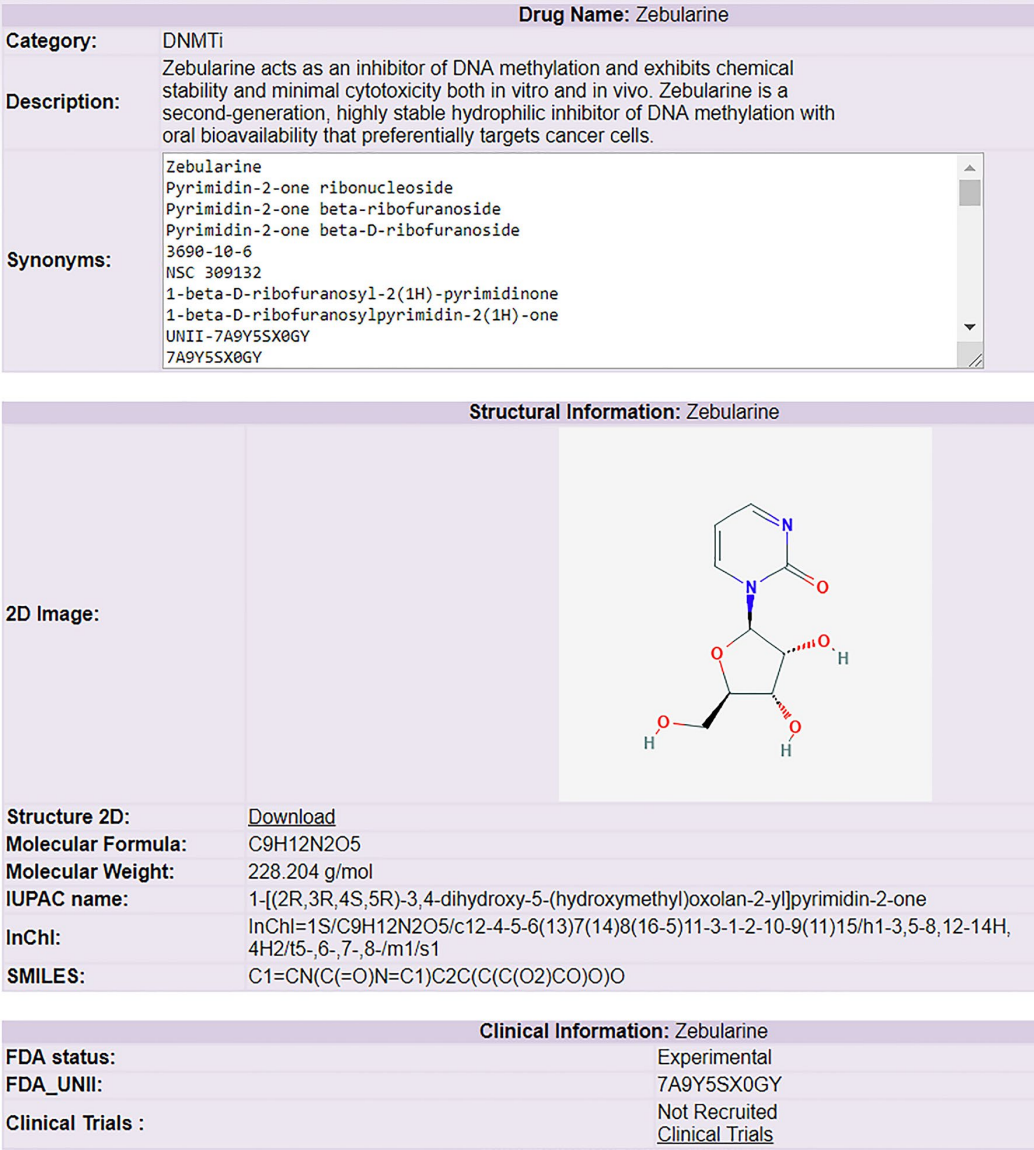
Representative image of epidrug ‘Zebularine’, a DNA methyltransferase inhibitor: the entry of ‘Zebularine’ epidrug is divided into multiple pieces of information like basic, structural, clinical, bioassay and references in the database

The new inclusion, EpiDrug database, highlights the different types of inhibitors based on the chromatin-modifying enzymes that either ‘write’ or ‘erase’ the functional groups. The individual category summarizes chemical molecules and potential drugs that are either approved by the FDA or are currently being used in in vitro or pre-clinical experimental studies. A total of 200 molecules have been identified by searching PubMed and pharmaceutical websites (https://www.medchemexpress.com/Pathways/Epigenetics.html) which are categorized into 12 different types. The individual entries of these molecules have information regarding their structure, chemistry, bioassay, and current phase trial status with a link to the ClinicalTrial.gov website for detailed information (Fig. 3). Further, the database also provides information about basic molecular properties like weight, formula, etc. for each drug. Three different chemical descriptors have been provided for each compound: (i) International Union of Pure and Applied Chemistry (IUPAC), (ii) Canonical Simplified Molecular-Input Line-Entry System (SMILES) [36–38], and (iii) IUPAC International Chemical Identifier (InChI) [39–41]. Also, the bioassay information is linked to the PubChem Bioassay website using the ID (AID) of each assay for providing data related to pharmacology, patents, and bioactivities. Further, individual drugs have been linked to the different databases like ChEMBL [42], ZINC DB [43], Human Metabolome DB [44], LiverTox [45] and Small Molecule Pathway Database [46] to give added information about their structures, toxicity and the biological impact on different tissues of human body after consumption of the drug.

### Sequence alignment of histone isoforms and variants

**Fig. 4:**
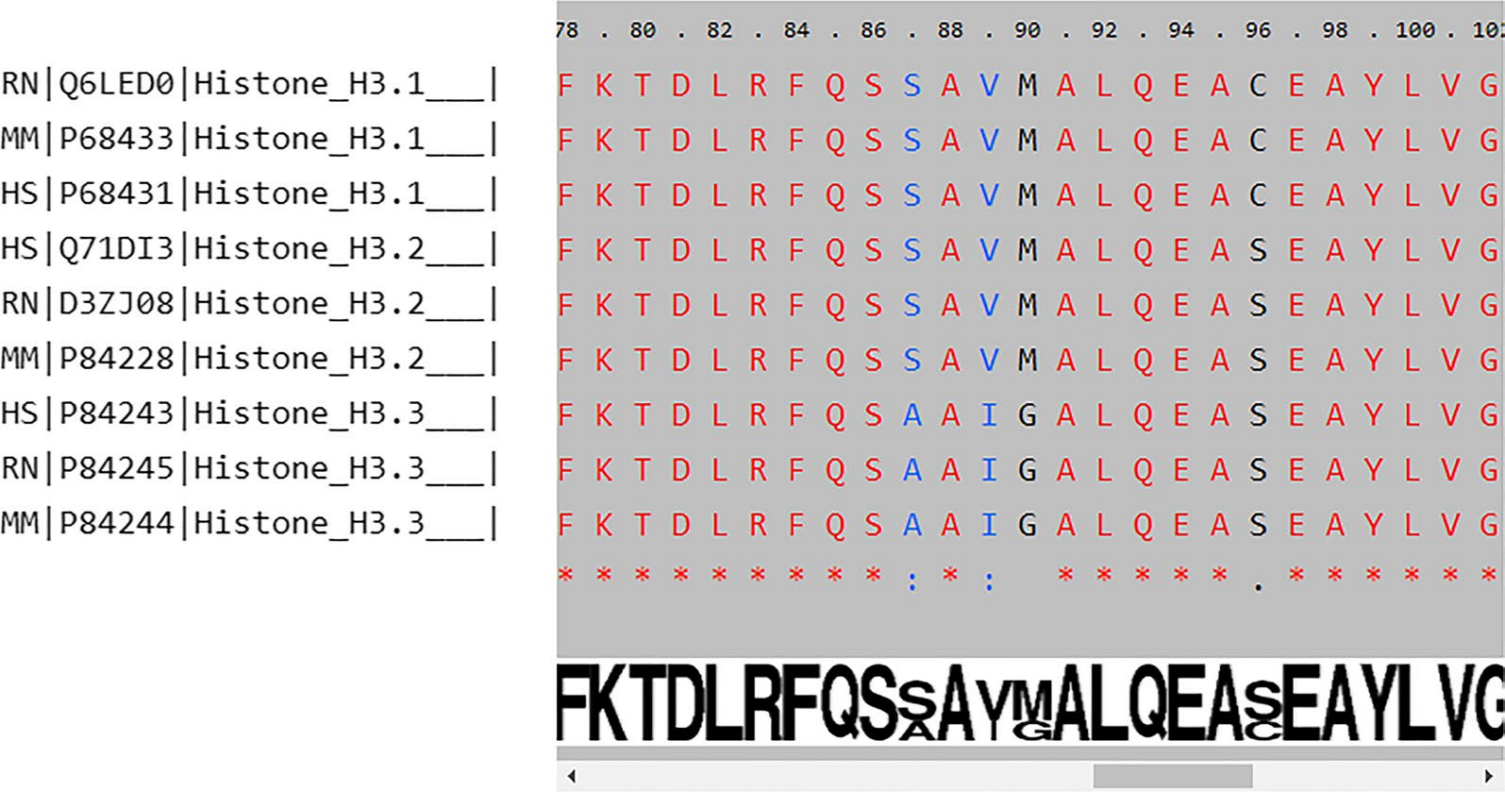
Representative image of sequence alignment of histone H3 proteins from human, mouse, and rat: the amino acids in ‘red’ color indicate identical residues in all proteins, ‘blue’ indicate amino acids substituted with similar properties among the queried proteins, while ‘black’ indicate amino acids with different properties among proteins. A ‘WebLogo’ is placed parallel below the alignment to highlight the relative frequency of occurrence

Multiple Sequence Alignment helps in aligning different proteins based on sequence similarities. The sequence alignment page displays a list of various histone variants and isoforms in human, rat, and mouse. The user can select single or multiple histone proteins from a single organism using the check-boxes or can compare protein sequences across the three species by selecting specific variants or isoform among the three organisms. For example, the output of multiple sequence alignment of histone H3 isoforms from human, mouse, and rat shows the favorable substitution position 87 (in blue); position 90 and 96 (in black) shows the unconserved region and identical amino acids are in red (Fig. 4). In continuation, the WebLogo indicates the overall height for the conserved amino acids, whereas the height at 87, 90 and 96 is adjusted based on the relative frequency of occurrence in an alignment. Histone isoforms within species and across species are quite similar. They differ by a few amino acids (1–3) within species. Therefore, MSA will provide information about the conservation of protein within and across species. The presence of specific amino acids in a protein sequence gives rise to specific secondary or tertiary structures. Even a single unfavorable amino acid substitution can disrupt the stability of the protein structure. Hence, studying regions of favorable substitutions, mutations, and conservations in the amino acid sequence become necessary to understand its importance in determining the protein’s structural integrity and its functional impact. The difference in the amino acid sequence could be the possible reason for structural and functional variability among the different histone isoforms. Also, based on the algorithm, one can predict the phylogenetic distance between the species using a given histone protein sequence. Therefore, with the help of the sequence alignment tool, researchers can study MSA of different histone orthologs from the given three organisms and explore their evolutionary relationship. The tool provides a pictorial representation of regions of conservation and substitutions, which could help researchers in studying variation among histones.

### Histone dimer stability prediction for histone H2A and H2B isoforms

In nucleosome organization, the wrapping of DNA with histone dimer of H2A/H2B and tetramer of H3/H4 plays a vital role in the formation of a stable nucleosome. Earlier studies have shown that differential incorporation of histone variants leads to changes in nucleosomal architecture [47]. The histone variants are not included in the present versions of the database for calculating energy minimization, as literature is available about their role in the stability of nucleosomes [47]. Histone H2A and H2B have multiple isoforms, H3 has two isoforms and H4 has none in humans [7]. The histone isoforms and their role in nucleosomal stability have not been studied in-depth; therefore, in the updated version, information related to the potential energy of H2A/H2B isoform dimers is included to predict the differences in the stability of nucleosome. The outputs are represented as a heatmap of histone H2A–H2B dimer with their potential energy. Earlier studies from our lab have shown that the nucleosomal stability of H2A1H/H2B was different compared to H2A.2/H2B, in silico as well as in vitro [48, 49]. At the dimer level also the stability was different for both the isoforms.

## Conclusion and future strategies

*HISTome2* is a freely available web-based resource that provides comprehensive information on histone isoforms, variants, histone PTMs, modifying enzymes, and epidrugs against ‘erasers’ and ‘writers’ and their pre-clinical as well as clinical trial status on a common platform. Histone isoforms and variants can also be compared across multiple organisms to understand their evolution/orthologs in other species. The common platform provides scientists to explore histone entries between and across organisms, their expression in cancer and potential miR targets. The inclusion of epidrugs against epigenetic modifiers on a common platform with histone-modifying enzymes will significantly enhance the possibility of successful planning of the pre-clinical and clinical research. The entries are connected with a peripheral link to different freely available databases to retrieve detailed and real-time updated information. The information on all these epigenetic factors in other species is also emerging rapidly. Thus, we intend to update the current database along with the integration of information for other eukaryotic organisms like yeast, *C. elegans,* and zebrafish in the future.

## Methods

### Constructions and content of *HISTome2*

#### Home page for integrative search

The interface and visualization of the *HISTome2* are developed using XHTML, JavaScript, PHP, and MySQL. The webpages of the *HISTome2* are dynamically loaded using AJAX and JavaScript functions, which are processed by PHP to present the data from MySQL. The schema of the database and connections between the tables is shown in Fig. 1. The information on human, mouse and rat database is stored in histone, PTM and enzyme tables which has a common schema to perform an integrative search on single keyword input. Whereas, the data on epidrugs are stored in drug info and bioassay tables which are connected by primary key ‘CID’. The data are stored in an MySQL relational database with 3-tier architecture viz. database, user and application tiers. The database tier contains the data stored in the form of tables; the user tier provides access to a user interface. The data in the application tier are accessed by Clustal Omega [50] and WebLogo [51].

#### Data mining and links to peripheral resources

In *HISTome2,* the information on histone proteins, PTMs and modifying enzymes in human, rat and mouse was manually curated from NCBI, uniport, gene-cards, histone DB2.0, and Talbert et al. [52–56]. The methodology for data mining for ‘detailed information page’ was modified from the earlier version (HIstome) because for chromosomal location, URL was no longer available and the Unigene database will be discontinued shortly. Therefore, UniProt accession ID was provided for all the entries as it is the most comprehensive protein database which provides detailed functional information for the proteins. Further, gene-related information such as name, symbol, and GeneID was acquired from HGNC [57], RGD [58], and MGI [59] for human, rat, and mouse, respectively. Gene, transcript, and protein-related information were obtained from NCBI, while EC number was fetched from EC-PDB [60]. Promoter sequences for different histones and modifying enzymes were obtained from EPD [61]. The PubMed link was provided to each entry with pre-embedded aliases in keywords to fetch updated information and to exclude non-specific searches. However, if the PubMed search did not display any literature, a specific PMID reference link is provided. An additional hyperlink was provided to all the entries for humans to retrieve TCGA mRNA expression from FireBrowse [33] and mIR targets from TargetScan [34].

A new component in the database, EpiDrug is added to highlight its importance in the field of epigenetics and potential in the treatment of different diseases. The information on the epidrugs was retrieved from PubMed, PubMed Central, and Google scholar, using different searches related to DNA methylation and histone-modifying enzymes like DNA methyltransferase inhibitors, histone acetyltransferase inhibitors, histone deacetylase inhibitors, histone methyltransferase inhibitors, histone acetyltransferase inhibitors, histone demethylase inhibitors, inhibitors of proteins binding to methylated histones, protein arginine deiminases inhibitors, poly (ADP-ribose) polymerase inhibitors, inhibitors of bromodomain (BRD) and extra-terminal domain (BET) family of proteins, and inhibitors of ubiquitinases and deubiquitinases. The information on synonyms, molecular formula, molecular weight, IUPAC, InChl, smiles, and 2D structures of epidrugs are obtained from the PubChem compound database [62]. The list of biological assays that have been performed on these chemical compounds to determine the chemical toxicity and bioactivity is acquired from the PubChem BioAssay database [2]. The information on the FDA status and ongoing clinical trials is fetched from ClinicalTrial.gov [63].

### Data repository and analysis tools

#### Sequence alignment

To elucidate the role of conserved amino acids in different types of histones isoforms and variants within an organism and across the three organisms, the sequence alignment tool is introduced in *HISTome2.* The selected protein sequences are processed using AJAX script and serve as an input to Clustal Omega for sequence alignment and return the complete output as a string [50]. Clustal Omega was run with default parameters such as gap penalty 10, gap extension penalty 0.20 and GONNET protein weight matrix. The alignment output file is handled using PHP scripts by displaying the alignment in a single-line scrollable window with the number assigned to amino acids in an alignment. The conserved residues (identities) in an alignment are marked with an asterisk in red color and while conservative substitution (similar) residues displayed with a colon in blue color. The same alignment file (output of clustal omega) is given as an input to the WebLogo tool to generate WebLogo image, which is aligned with the Clustal Omega output using javaScript [51].

#### Histone dimer stability prediction for histone H2A and H2B isoforms

The binding affinity between histone dimers dictates the stability of the nucleosome [49]. The PDB ID: 2CV5 was taken as a template structure for building histone H2A and H2B isoforms dimer of human, mouse, and rat using Discovery studio visualizer. The Gromacs 2018 was used to calculate the potential energy of the dimers [64]. The dimers were solvated with TIP3P water molecules in a cubic box with periodic boundary conditions of 1.0 nm from the edge of the box [65]. Since the histones are rich in positively charged residues, the overall positive charge of the system was neutralized by adding chlorine as counter ions. The systems were energy minimized by the steepest descent algorithm with the tolerance of maximum force of 1000.0 kJ/mol/nm, implementing OPLS-AA force field [66]. The potential energies values of the dimers were clustered using the R package ‘gplots’ to plot the heat-maps of histone H2A and H2B isoform dimers of human, rat, and mouse.

## Key points

- *HIST*ome2 provides in-depth information on histone proteins (variants or non-canonical and isoforms or canonical), PTMs and modifying enzymes for human, mouse, and rat on a single platform.
- It also provides detailed information on epidrugs for various categories HATi, HDACi, DNMTi, etc. to highlight their importance in the treatment of different diseases.
- Overall, the *HIST*ome2 has significantly increased the extent and diversity of its content and thus assisting in the search and comparative interpretation of the multifactorial parameters in the field of histone biology.
- The database will serve as a useful “knowledge base” for basic researches, clinical scientists, pharmacologists in understanding the histone biology and their potential importance in epidrug discovery through its ability to connect chemistry, biology, and informatics.
- *HIST*ome2 is freely available at http://www.actrec.gov.in/histome2/.

## Data Availability

*HIST*ome2 data are available at http://www.actrec.gov.in/histome2/
